# A new look at Hsp70 activity in phosphatidylserine-enriched membranes: chaperone-induced quasi-interdigitated lipid phase

**DOI:** 10.1038/s41598-023-46131-x

**Published:** 2023-11-06

**Authors:** Ruslana Tagaeva, Svetlana Efimova, Alexander Ischenko, Alexander Zhakhov, Maxim Shevtsov, Olga Ostroumova

**Affiliations:** 1 Personalized Medicine Centre, Almazov National Medical Research Centre, Akkuratova Str. 2, Saint Petersburg, 197341 Russia; 2 Institute of Cytology of the Russian Academy of Sciences (RAS), Tikhoretsky Ave. 4, Saint Petersburg, 194064 Russia; 3 Saint-Petersburg Pasteur Institute, Mira Str. 14, Saint Petersburg, 197101 Russia; 4 Department of Radiation Oncology, Technishe Universität München (TUM), Klinikum rechts der Isar, Ismaninger Str. 22, 81675 Munich, Germany

**Keywords:** Biochemistry, Cell biology

## Abstract

70 kDa heat shock protein Hsp70 (also termed HSP70A1A) is the major stress-inducible member of the HSP70 chaperone family, which is present on the plasma membranes of various tumor cells, but not on the membranes of the corresponding normal cells. The exact mechanisms of Hsp70 anchoring in the membrane and its membrane-related functions are still under debate, since the protein does not contain consensus signal sequence responsible for translocation from the cytosol to the lipid bilayer. The present study was focused on the analysis of the interaction of recombinant human Hsp70 with the model phospholipid membranes. We have confirmed that Hsp70 has strong specificity toward membranes composed of negatively charged phosphatidylserine (PS), compared to neutral phosphatidylcholine membranes. Using differential scanning calorimetry, we have shown for the first time that Hsp70 affects the thermotropic behavior of saturated PS and leads to the interdigitation that controls membrane thickness and rigidity. Hsp70-PS interaction depended on the lipid phase state; the protein stabilized ordered domains enriched with high-melting PS, increasing their area, probably due to formation of quasi-interdigitated phase. Moreover, the ability of Hsp70 to form ion-permeable pores in PS membranes may also be determined by the bilayer thickness. These observations contribute to a better understanding of Hsp70-PS interaction and biological functions of membrane-bound Hsp70 in cancer cells.

## Introduction

Heat shock protein 70 (Hsp70) is a highly conserved member of the 70 kDa chaperone family HSP70 that performs many critical functions in both eukaryotic and prokaryotic cells. The HSP70 family includes at least eight members that differ in amino acid residues in their domains and have some tissue-specific expression^1^. Hsp70 protein, also called HSP70A1A, consists of two domains: an N-terminal ATP-dependent nucleotide-binding domain (NBD) and a C-terminal substrate-binding domain (SBD), coupled with a flexible linker. It is a stress-inducible protein synthesized by the cell under stressful conditions (e.g., heat shock, hypoxia, ionizing radiation, oxidative stress, etc.). The functions of the Hsp70 in eukaryotic cells are quite diverse including the folding/refolding of misfolded proteins and polypeptides, protein translocation and degradation, regulation of the processes of apoptosis, ferroptosis, and autophagy^2–5^.

In addition to intracellular localization, Hsp70 is present on the plasma membranes of various solid and hematological malignancies, but not on the membranes of normal cells^6–8^. The exact mechanisms of Hsp70 anchoring to the lipid bilayer and protein functions are still not fully understood. The investigation of Hsp70 interaction with membrane components, its incorporation into lipid bilayer and externalization on the membrane surface is sophisticated due to the fact that protein does not contain any known transmembrane sequence. It is assumed that Hsp70 is transferred to the plasma membrane via non-classical vesicular mechanisms, since inhibitors of the classical ER-Golgi pathway do not affect membrane-bound Hsp70 (mHsp70) expression^9^.

Currently, several lipid components of the plasma membrane of tumor cells have been proposed as partners of Hsp70 for its anchoring on the membrane. One potential partner is the glycosphingolipid globoyltriaosylceramide (Gb3). Gb3 is a component of cholesterol-enriched lipid domains (termed lipid rafts) overexpressed in the membranes of tumor cells^10–12^. Staining of Gb3 and mHsp70 on tumor cells revealed their colocalization employing confocal microscopy^13^. Treatment of tumor cells with methyl-β-cyclodextrin, that preferentially affects cholesterol, led to a significant decrease in mHsp70 expression, which is consistent with previous assumptions about the presence of Hsp70 in lipid rafts^14,15^. Moreover, in vitro experiments with liposomes confirmed that Hsp70 predominantly binds to vesicles containing Gb3^13^.

In addition to Gb3, Hsp70 is able to interact with negatively charged phosphatidylserine (PS) on the plasma membrane of tumor cells^16,17^. Under non-stress conditions, PS is located in the inner leaflet of the lipid bilayer of normal cells, but under the influence of various stress factors, it moves to the outer leaflet through the activation of the Ca^2+^-dependent phospholipid scramblase and reduced the ATP levels, leading to a loss of PS asymmetry in the membrane^18^. Non-apoptotic tumor cells contain elevated level of surface PS regulated by differential flippase activity and intracellular calcium^19^. Hypoxia treatment of tumor cells causes colocalization of Hsp70 and PS on the cell surface, which is also observed when an exogenous protein is added to normoxic and hypoxic tumor cells^17^. Bilog et al.^16^ further proved the binding Hsp70 and PS localized in cytoplasmic leaflet of the membrane of tumor cells. This is consistent with the hypothesis that Hsp70 can be transferred through the membrane by flipping of PS from the inner to the outer leaflet^20,21^. This process may be followed by the rapid return of PS to the inner side of membrane by flippase, leaving the protein embedded in the bilayer^22^.

The specific interaction between PS and recombinant Hsp70 was also shown using liposomes of various compositions^17,23–28^, while interaction with neutral lipids such as phosphatidylcholine (PC) was weak and unspecific^27,29^. Hsp70, but not Hsp90, was able to selectively integrate into unilamellar palmitoyloleoylphosphatidylserine (POPS) vesicles, and this incorporation was proportionally reduced by decreasing the content of POPS^25^. Presumably, electrostatic Hsp70-lipid interactions are required only for the initial association with the plasma membrane, then anchoring is associated with the alignment of protein domains with acyl chains of dipalmitoylphosphatidylserine (DPPS)^26,30^. In support of the assumption, the association of Hsp70 with model membranes is enhanced by increasing the saturation level of PS fatty acid chains^26,29^. Alder et al.^31^ also reported that Hsp70 promotes the leakage of calcein from liposomes composed of mixture of soy phospholipids (neutral PC and phosphatidylethanolamine, and negatively charged phosphatidylinositol). This is most likely due to the ability of Hsp70 to form pores in the negatively charged lipid bilayers^21,32^. Another constitutive member of the HSP70 family, heat shock cognate 71 kDa protein (Hsc70) is capable of forming very stable and uniform ion conductance channels, and activity of these channels are regulated by the presence of ATP/ADP^33^. Moreover, Hsc70 and Hsp70 are able to aggregate POPS liposomes in a time- and concentration-dependent manner, which also depends on the content of ATP/ADP^23^.

In the current study we aimed to identify the lipid determinants for Hsp70 action on model membranes. The protein impacted thermotropic behavior of PS by inducing interdigitation. Hsp70 effects on domain organization of PS-enriched membranes was dependent on the PS acyl tail saturation. A dependence of the Hsp70 pore-forming ability on the PS content and the lipid bilayer thickness was demonstrated.

## Results and discussion

### Hsp70 induces the quasi-interdigitated phase in the membranes composed of phosphatidylserines

**Figure 1:**
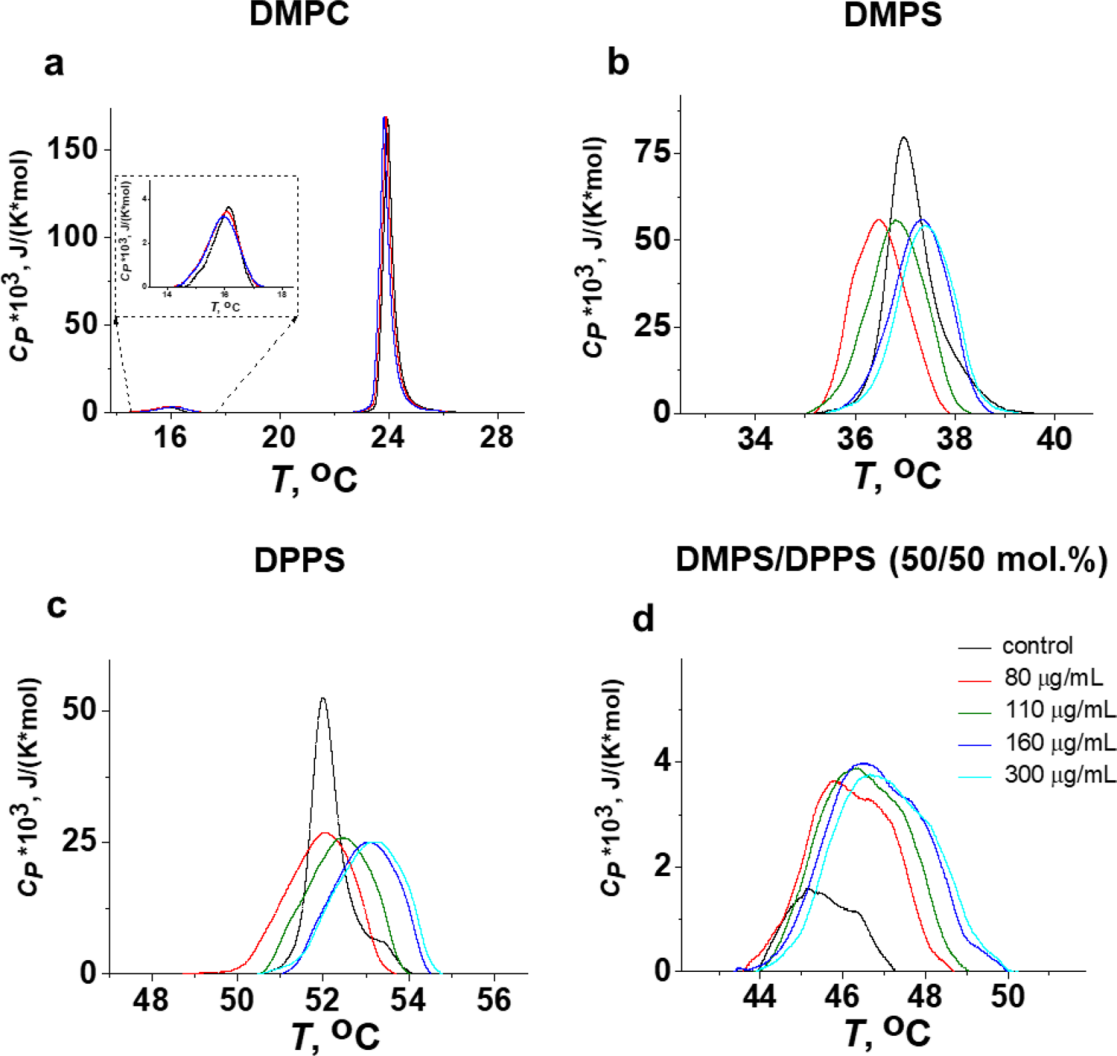
Heating thermograms of DMPC (**a**), DMPS (**b**), DPPS (**c**), and equimolar mixture of DMPS/DPPS (50/50 mol.%) (**d**) in the absence (control, black curves) and presence of Hsp70 at concentration of 80 (red curves), 110 (green curves), 160 (blue curves) and 300 μg/mL (cyan curves).

We studied the effect of Hsp70 on the thermotropic behavior of the neutral DMPC and negatively charged DMPS using differential scanning microcalorimetry. Figure 1a demonstrates the heating thermograms of DMPC in the absence (control, *black curve*) and in the presence of Hsp70 at concentrations of 80 (*red curve*) and 160 μg/mL (*blue curve*). Hsp70 did not affect the main thermotropic parameters of the DMPC at both concentrations (Δ*T*_*m*_, Δ*T*_*1/2*_, and ΔΔ*T*_*h*_ is about zero), confirming no interaction between protein and PC.

**Figure 2:**
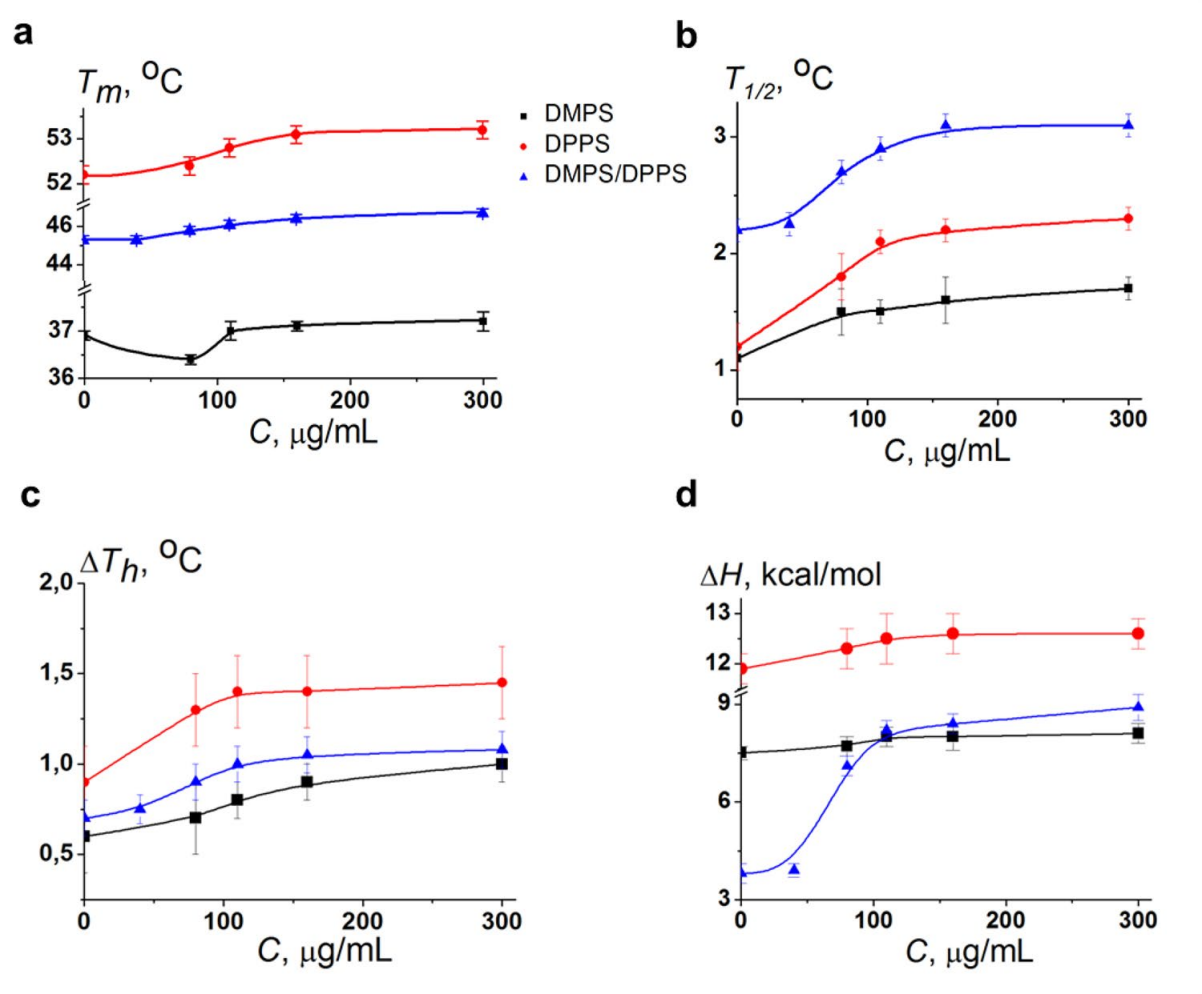
The dependence of the parameters characterizing the thermotropic behavior of DMPS (black curves), DPPS (red curves), and equimolar mixture of DMPS/DPPS (50/50 mol.%) (blue curves) on Hsp70 concentration: (**a**) the melting temperature of heating scan (*T*_*m*_); (**b**) the half-width of the main peak (*T*_*1/2*_), (**c**) the difference in the transition temperatures between heating and cooling scans (*T*_*m*_-hysteresis, Δ*T*_*h*_), and (**d**) transition enthalpy (Δ*H*).

**Figure 3:**
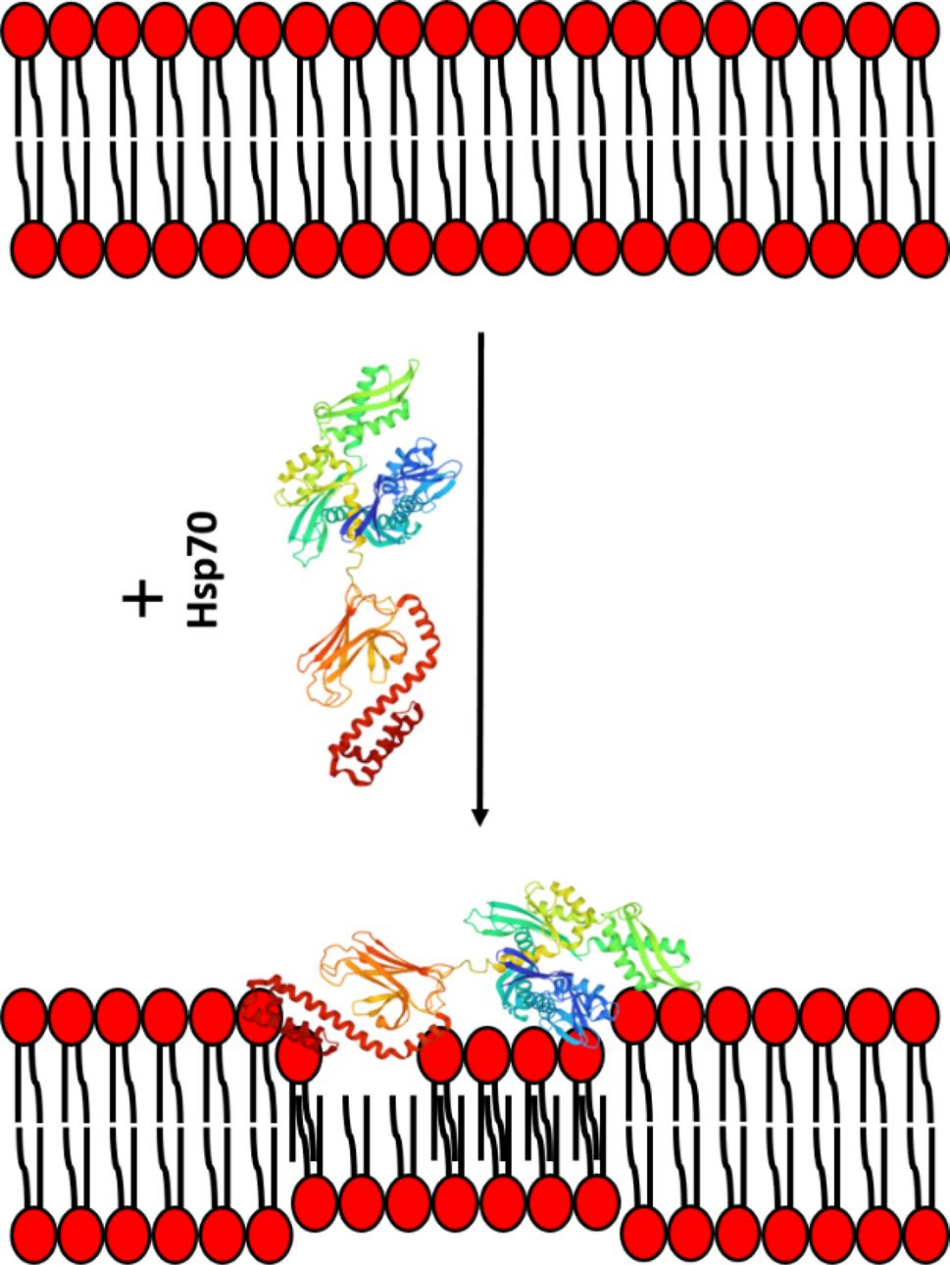
Schematic representation of Hsp70 induction of the quasi-interdigitated phase in the membranes composed of PSs. Hsp70 atomic structure has a PDB code 2KHO; negatively charged PS molecules are shown in red.

Figure 1b and Supplementary Table S1 demonstrates that Hsp70 was characterized by dramatic effects on the thermotropic behavior of DMPS, indicating a strong interaction of the protein with bilayer composed of negatively charged PS. The biphasic phase behavior of DMPS was observed in the presence of protein at various concentration: an addition of Hsp70 up to 80 μg/mL led to decrease in transition temperature (*T*_m_), a subsequent increase in Hsp70 concentration up to 110 μg/mL caused an enhancement in *T*_m_-value, which was practically stabilized at concentrations more than 110 μg/mL (Fig. 2a, Supplementary Table S1). Moreover, Fig. 2 shows that the biphasic behavior was also reflected in the increase in the half-width of the DMPS transition peak (*T*_1/2_, b), the difference in the *T*_m_-values between heating and cooling scans (*T*_m_-hysteresis, Δ*T*_*h*_, c) and the transition enthalpy (Δ*H*, d) as the protein concentration increased (Supplementary Table S1). The observed biphasic effects might indicate that Hsp70 induces quasi-interdigitated phase (Fig. 3). It should be noted that Hsp70 did not practically affect the thermotropic behavior of TMCL and DPPG (Supplementary Fig. S1 and Table S2) that was consistent to the assumption that lipid interdigitation is due to specific interaction of the protein with the PS and it was not a result of nonspecific electrostatic interaction of Hsp70 with the anionic phospholipids.

Interdigitation is a rearrangement of lipid molecules in bilayer, when the hydrophobic tails of the phospholipids from opposite membrane leaflets interlock and interpenetrate. This leads to a decrease in the membrane thickness, an increase in the membrane packing density, a change in surface electrostatic properties and interleaflet coupling^34^.

It is well known that the propensity of saturated PCs to interdigitate under chemical inducers enhances as the hydrocarbon chain length increases^35^. We hypothesized that DPPS having the longer acyl chains (16:0) compared to DMPS (14:0) would be easier to interdigitate upon Hsp70 addition, i.e. dependence of thermotropic characteristics on protein concentration will shift horizontally towards lower concentrations. In confirmation, the introduction of protein to a concentration of 110 μg/mL led to significant increase in *T*_m_-value of DPPS (Fig. 2a, *red curve,* Supplementary Table S1) contrast to inefficiency of this concentration in the case of DMPS (Fig. 2a, *black curve,* Supplementary Table S1). The mixing short-tailed and long-tailed lipids should also promote interdigitation. The Hsp70-induced interdigitation in the equimolar mixture of DMPS/DPPS is clearly demonstrated by a dramatic increase in the enthalpy of transition with increasing protein concentration (Figs. 1d, 2d, Supplementary Table S1). Moreover, our assumption is consistent with the studies of Lamprecht et al.^26^.

Based on results, we suggest that, when inserted into the membrane, Hsp70 is able to induce interdigitation, thereby leading to a decrease in membrane thickness and an increase in the lipid packing density. Potentially, a protein could use this ability to facilitate membrane pore formation by adjusting the surrounding lipid bilayer to its own three-dimensional structure to decrease hydrophobic mismatch. Moreover, Hsp70-induced membrane interdigitation might also impact to resistance to anti-cancer therapy due to larger membrane rigidity and decreased permeability. Indeed, the accumulating evidence implicate that the HSPs stabilization of biological membranes might be linked to the cancer cells primary stress-sensing mechanisms^36^. In the observation by Tsuchiya et al.^37^ it was revealed that several agents including epigallocatechin gallate, resveratrol, genistein, apigenin and anticancer drug doxorubicin significantly rigidified the tumor cell model membranes, particularly in the hydrocarbon core than the membrane surface. This in turn influences the cancer cells sensitivity towards doxorubicin^38^. However, it is still an open question whether changes in the lipid composition and/or concomitant conformational changes of proteins anchored in the membrane directly lead to chemoresistance of cells. Thus, P-glycoprotein that effluxes drug molecules has been demonstrated to be highly sensitive to the changes of membrane rigidity^39^.

There are studies indicating the ability of some proteins to change the *T*_*m*_ of lipids and lead to membrane remodeling. For example, Nogo-66, which is a part of the extracellular domain of the neurite outgrowth inhibitor protein, is capable of inducing interdigitation in DMPC membranes^40,41^. Another protein which is believed to be involved in the rearrangement of the PS-enriched lipid bilayer is α-synuclein, is a presynaptic neuronal protein whose aggregation is related to the Parkinson’s disease^42,43^. High concentration of α-synuclein induces lateral expansion of lipid molecules, positive curvature in bilayer and membrane thinning via interdigitation^44^. Moreover, it has been shown that when bound to the membrane, α-synuclein is able to permeabilize lipid bilayer through the formation of pores or conductive ion channels^45–48^. Pore formation was observed both for the monomeric form of α-synuclein and for oligomers in negatively charged membranes composed of PS, phosphatidylglycerol, and phosphatidic acid, but not in neutral ones. It was found that some peptides, such as human antimicrobial peptide cathelicidin (LL-37), frog-skin antimicrobial peptide peptidyl-glycylleucine-carboxyamide, and N-terminal domain of the capsid protein cleavage product of the flock house virus, induce the formation of the interdigitated gel phase in lipid bilayers with different composition^49–52^.

An intriguing example of a membrane-active protein is Hsp12 from the yeast *S. cerevisiae*. Welker et al.^53^ showed that Hsp12 binds selectively to a negatively charged lipids such as phosphatidylglycerol and phosphatidylinositol, comparing with a positive charged or neutral one. Moreover, Hsp12 is able to alter the physicochemical characteristics of the membrane composed of phosphatidylglycerol, leading to the formation of a ripple phase structures. A possible mechanism for the effect of membrane-active proteins on the phase behavior of lipids is that proteins recognize lipid-packing defects and interacts with the membrane, which, in turn, rearranges its structure to maintain its own integrity^44,54^.

In cells, interdigitation can affect the processes of transduction of intracellular signals and the transport of molecules across the membrane. Mass spectrometry analyses of PC-3 prostate cancer cells and their released exosomes revealed a strong interdigitation between stearoyloleoylphosphatidylserine in the inner leaflet and the very-long-chain sphingomyelin species in the outer leaflet^55^. This cross-linking of lipids may contribute to PS clustering and influence on some functions of PS-binding proteins such as K-Ras, Rho GTPase Cdc42, Akt, etc.^56–58^. It has previously been reported that interdigitation occurs in membrane domains with a high lipid order and it may play a role in toxin-induced signaling^59–61^. For instance, it is assumed that bacterially produced Shiga toxin, when bound to Gb3 located in lipid rafts, can promote membrane interdigitation, leading to the interleaflet coupling between Gb3 and lipids from inner leaflet (potentially PS), which, in turn, transmit a signal to the cytoplasm. To deep insight the molecular mechanisms of Hsp70-PS interaction we examined the effects of PS-enriched ordered and disordered lipid domains.

### Hsp70 potentiates ordered negatively charged domains

**Figure 4:**
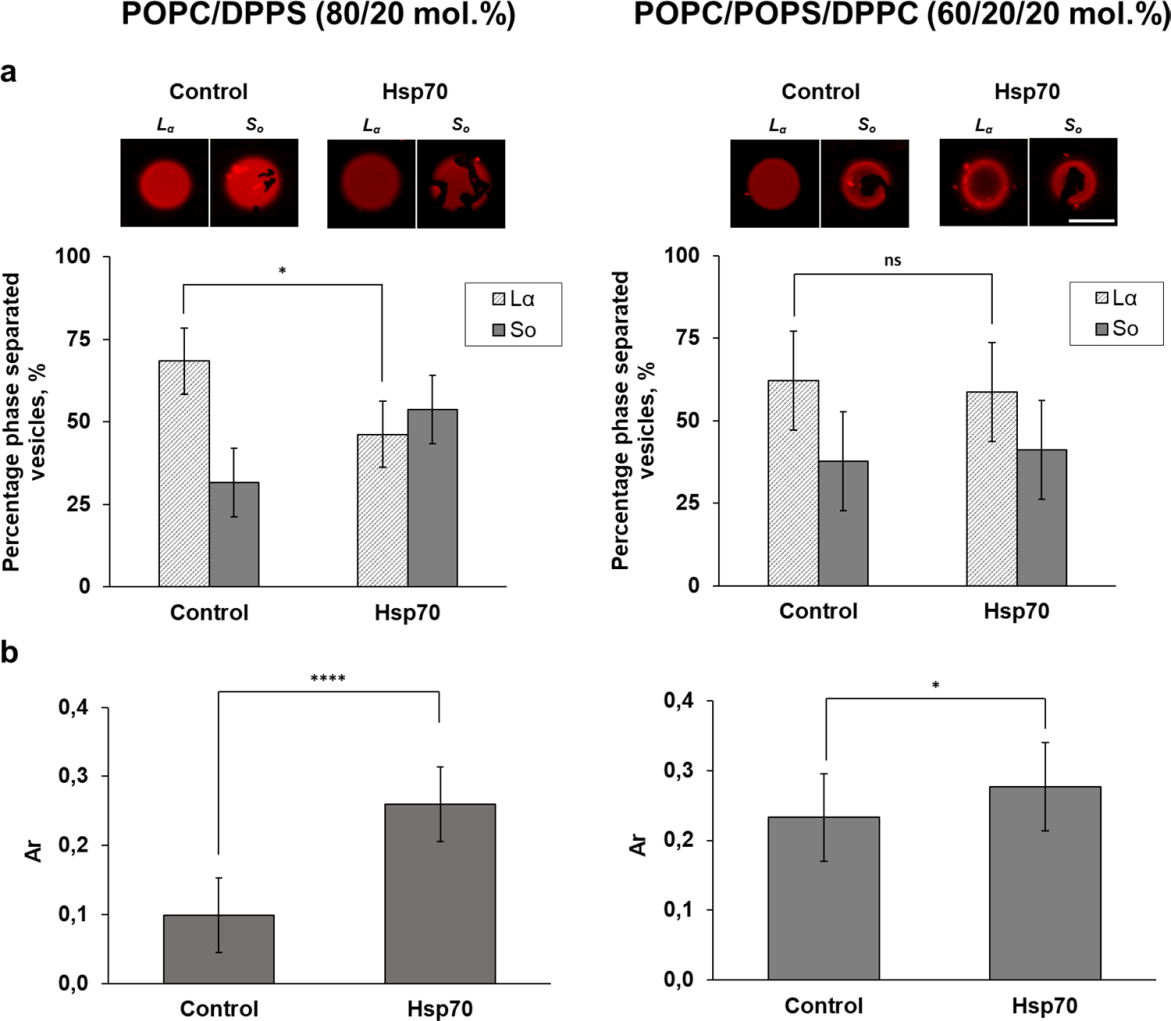
Diagrams of percentage of phase separated vesicles with typical GUVs confocal micrographs (scale bar is equal to 10 µm) (**a**) and *A*_*r*_ (the ratio of the *S*_*o*_ domain area to the total GUV area) (**b**) in POPC/DPPS (80/20 mol.%) and POPC/POPS/DPPC (60/20/20 mol.%) samples before (control) and after Hsp70 addition. At least 10 experiments were performed for counting of percentage of phase separated vesicles, 150 ± 15 images were processed for *A*_*r*_ calculating. The significance is represented as ^*ns*^*p* ≥ 0.05, *p ≤ 0.05, **p ≤ 0.01, ***p ≤ 0.001, and ****p ≤ 0.0001.

**Figure 5:**
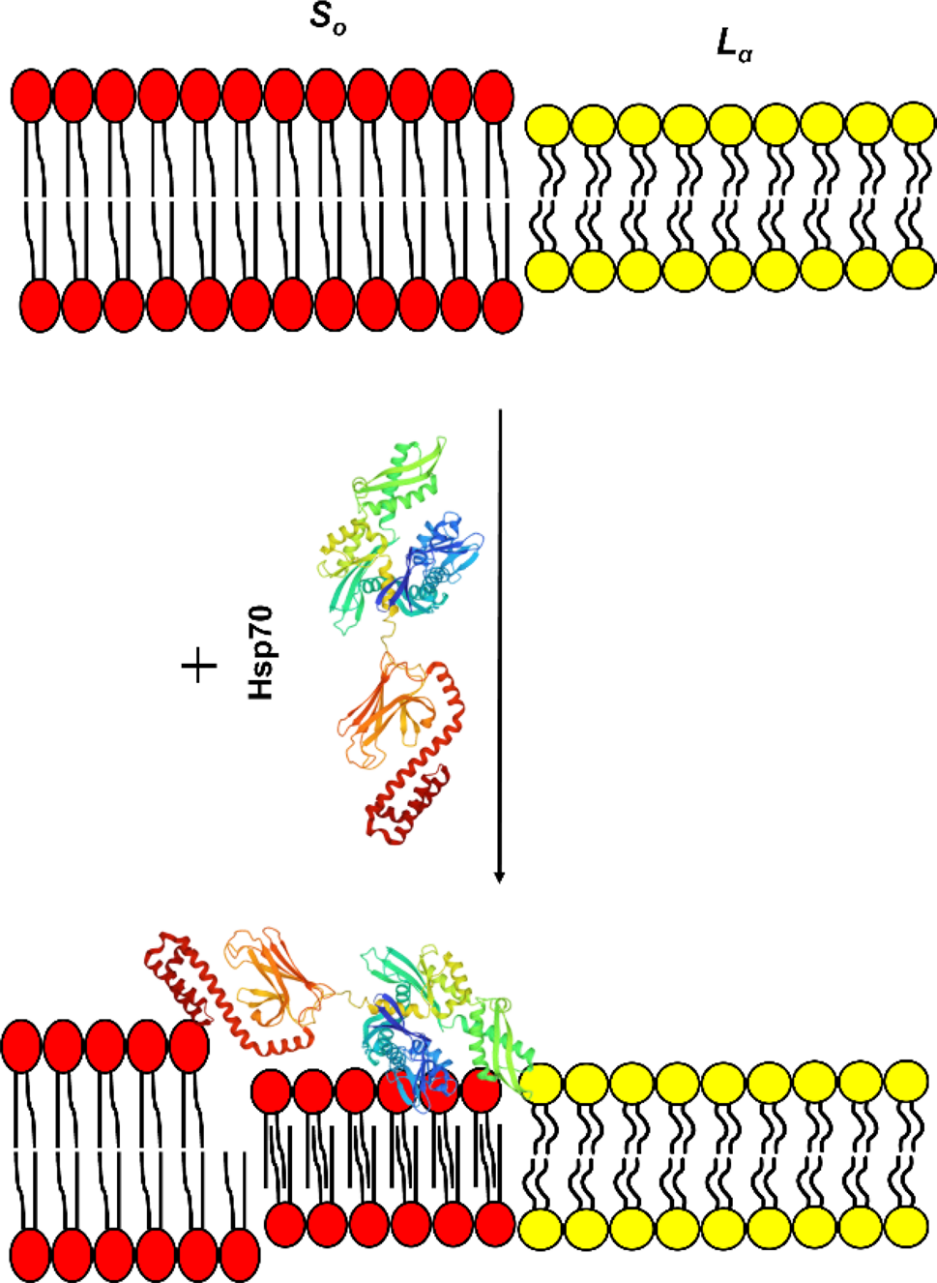
Schematic representation of the Hsp70 ability to reduce hydrophobic mismatch and facilitate formation of ordered domains via interdigitation. Hsp70 atomic structure has a PDB code 2KHO; negatively charged PS molecules in ordered lipid phase (*S*_*o*_) are shown in red, neutral PC molecules in disordered domains (*L*_*α*_) are shown in yellow.

Confocal fluorescence microscopy was employed to detect the interaction of Hsp70 with PS in giant unilamellar vesicles (GUVs) composed of POPC/DPPS (80/20 mol.%) and POPC/POPS/DPPC (60/20/20 mol.%). Rh-DPPE was used as a fluorescent label, which prefers the disordered domain (*L*_*α*_) and is excluded from the ordered lipid phase (*S*_*o*_)^62^. Figure 4a shows typical micrographs of GUVs made from respective lipid compositions in the absence (*control*) and presence of Hsp70, as well as a statistical description of their domain organization. Approximately 65% of liposomes composed of POPC/DPPS (80/20 mol.%) were homogeneously colored in the absence of Hsp70, while 35% of POPC/DPPS (80/20 mol.%) vesicles demonstrated uncolored domains of irregular shape that were organized by a negatively charged DPPS. The addition of protein led to about 20% decrease and appropriate increase in the relative number of homogeneously colored vesicles and liposomes with uncolored *S*_*o*_ domains respectively, which might indicate the ability of Hsp70 to integrate into DPPS-containing liposomes and to stabilize the DPPS-enriched ordered domains. This observation can be easily rationalized by the above assumption of protein-associated quasi-interdigitated DPPS phase (Figs. 1c and 2, Supplementary Table S1). The probability of ordered domain appearance is related to high energetic cost of formation of *S*_*o*_/*L*_*α*_ boundary due to a larger hydrophobic thickness of *S*_*o*_ domains than the surrounding *L*_*α*_ regions. Hsp70-induced DPPS interdigitation should lead to decrease in the hydrophobic mismatch and appropriate increase in the probability of ordered domain (Fig. 5).

Further we tested the ability of Hsp70 to influence phase separation in POPC/POPS/DPPC (60/20/20 mol.%) mixture, where POPS is located in a disordered liquid phase, while the ordered phase is predominantly formed by neutral DPPC. Taking into account that, the protein cannot bind DPPC (Fig. 1a) and the presence of one unsaturated acyl chain should inhibit the protein-induced interdigitation of POPS-enriched *L*_*α*_ regions, we expected that an addition of Hsp70 into solution bathing POPC/POPS/DPPC (60/20/20 mol.%) liposomes will not produce any noticeable effects. As expected, Fig. 4 shows that the addition of Hsp70 to vesicles composed of POPC/POPS/DPPC (60/20/20 mol.%) did not practically affect lipid phase separation.

To further demonstrate a higher probability of formation of ordered domains in the presence of Hsp70 in POPC/DPPS (80/20 mol.%) liposomes compared to its absence and inability of the protein to influence the lipid segregation in POPC/POPS/DPPC (60/20/20 mol.%) vesicles we compared how the area of ordered domains in POPC/DPPS (80/20 mol.%) and POPC/POPS/DPPC (60/20/20 mol.%) vesicles varied after protein insertion (Fig. 4b). Using the ImageJ software, we determined the ratio of area of all *S*_*o*_-domains to the total area of the liposome (*A*_*r*_). The results revealed that Hsp70 led to 2.6-fold increase in the *A*_*r*_ in POPC/DPPS (80/20 mol.%) but not in POPC/POPS/DPPC (60/20/20 mol.%) vesicles, that once again indicated the ability of protein to interact with ordered DPPS-enriched phase and protein inability to influence disordered POPS-containing domains. The results may imply that in cancer cells Hsp70 is able to interact with negatively charged saturated PSs in lipid rafts. The incorporation of Hsp70 into the membrane and facilitation of PS interdigitation in ordered domains might trigger signaling cascades and affect the transduction of intracellular signals. Several studies point to a possible location of Hsp70 in lipid rafts^13,14,63,64^, but its biological functions and the exact anchoring mechanism are not yet fully understood. To try to clarify this issue, in future studies we are going to investigate the interaction of the protein with various raft-associated lipids such as ceramides, sphingomyelin, and gangliosides.

### Hsp70 pore formation depends on the length of PS fatty acyl tails

**Figure 6:**
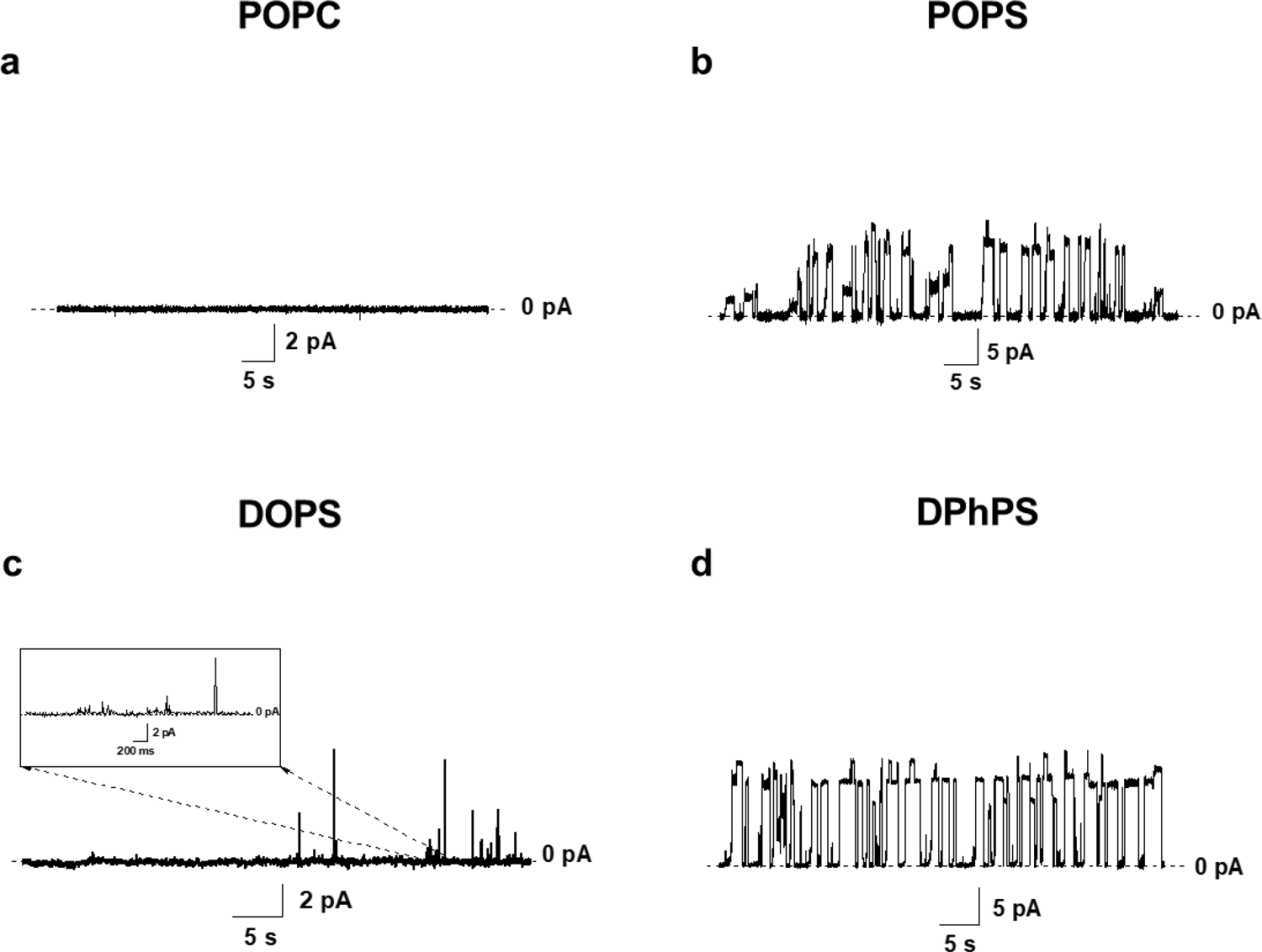
Examples of records of transmembrane current fluctuations induced by Hsp70 in lipid bilayers composed of POPC (**a**), POPS (**b**), DOPS (**c**), and DPhPS (**d**) and bathed in 0.1 M KCl pH 7.4. Protein concentration was 5.0 µg/mL (**a**), 2.0 µg/mL (**b**), 4.5 µg/mL (**c**), and 2.0 µg/mL (**d**). The transmembrane voltage was equal to 100 mV. One experiment out of 3 replicates with essentially the same results is shown.

The effects of Hsp70 on the ion conductance of planar lipid bilayers composed of neutral POPC and negatively charged POPS, DOPS, and DPhPS were tested. The addition of the Hsp70 up to the concentration of 5.5 µg/mL to neutral POPC membranes did not lead to increase in the ion permeability of lipid bilayers (Fig. 6a). The subsequent enlargement in the Hsp70 concentration caused the violation in the electrical stability of lipid bilayers and their subsequent destruction occurred. Table 1 summarized the parameters referred to the effects of Hsp70 on the properties of phospholipid bilayers. Replacement of the neutral POPC for the negatively charged POPS led to formation of Hsp70 induced transmembrane pores with different amplitudes at concentration of 2.0 ± 0.5 µg/mL. Figure 6b demonstrates the typical examples of current fluctuations corresponding to openings and closures of single pores induced by Hsp70 in POPS membranes. The amplitude of observed step-like current fluctuations varied in the range from 1 to 20 pA at the transmembrane voltage of 100 mV. The further increase in the Hsp70 concentration more than 3.5 µg/mL resulted in a membrane destruction. The observed decrease in the protein threshold concentration in the POPS membranes compared to POPC bilayers and the different pattern of the lipid-protein interplays indicated a specific interaction of Hsp70 with negatively charged POPS.

**Table 1:** The effect of Hsp70 on the ion permeability of phospholipid bilayers.

Lipid composition	*C*_*sc*_ (μg/mL)	*C*_*tr*_ (μg/mL)
POPC	*nf*	5.5 ± 0.5
POPS	2.0 ± 0.5	3.5 ± 0.5
DOPS	*nf*	4.4 ± 0.4
DPhPS	2.0 ± 0.2	3.5 ± 0.3

*C*_*sc*_ the threshold concentration of Hsp70 required to appearance of step-like current fluctuations corresponding to single ion-permeable transmembrane pores, *C*_*tr*_ the threshold concentration of Hsp70 required to disintegrate lipid bilayers of appropriate composition, *nf* single pores were not observed.

It is assumed that Hsp70 forms pores in the plasma membrane due to its ability to organize oligomers. In the absence of peptide targets and denatured proteins, Hsp70s tend to self-assemble to form low-order oligomers, regulated by ATP^65,66^. Apparently, after recognizing the negatively charged head groups of PSs, Hsp70 changes conformation, undergoes oligomerization, and is integrated into the lipid bilayer. It is considered that upon insertion, a fragment of the C-terminus of the SBD is exposed to the extracellular space, which was established using specific antibodies to various protein epitopes^67^. The N-terminus of Hsp70 is likely to remain in the cytoplasm, since the protein retains its nucleotide-binding function, and ATP/ADP regulate the opening and closing of ion channels^21,33^. The detected multilevel conductance of Hsp70-induced channels and different patterns of channel activity may indicate the incorporation of Hsp70 oligomers with different orders into the lipid bilayer. Clusters of β-sheets located in the SBD of Hsp70 may be responsible for oligomerization. So, β-amyloid peptides are able to form β-sheets that integrate into the membrane to form ion channels^68,69^.

**Figure 7:**
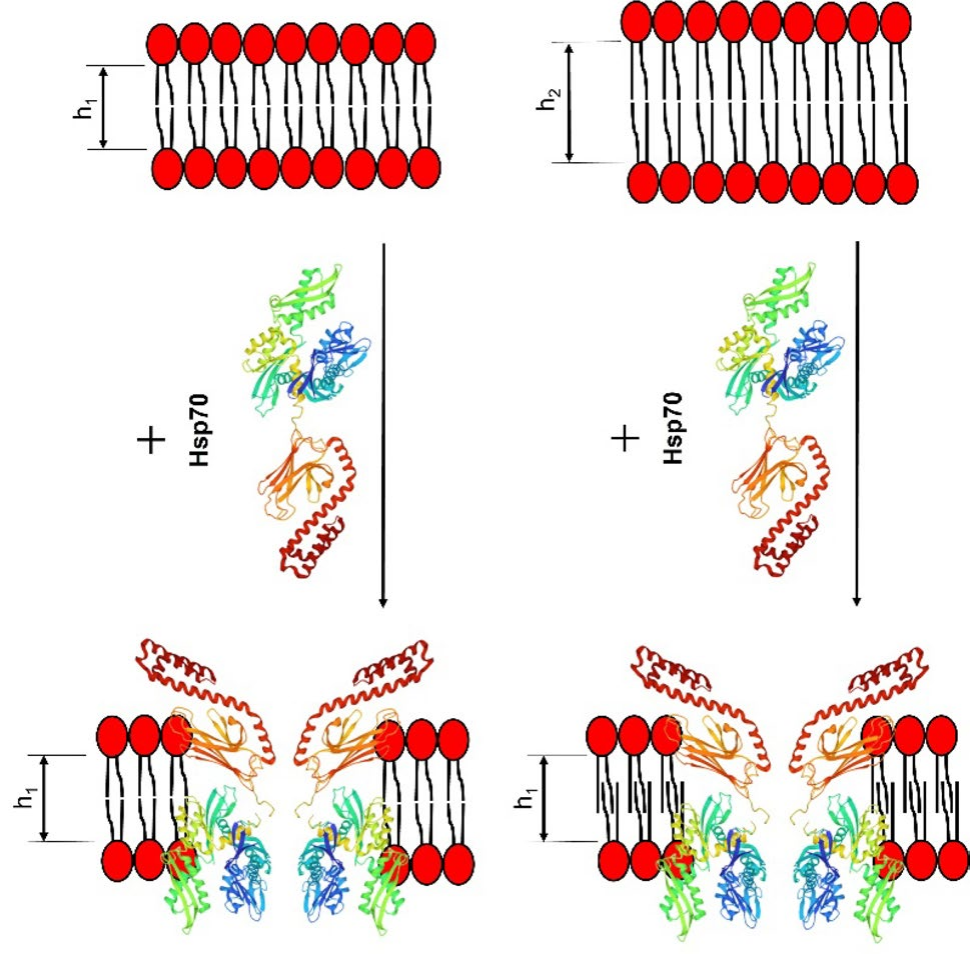
Schematic representation of the Hsp70 pore formation in PS-enriched membranes with different thicknesses (h_2_ > h_1_). Hsp70 atomic structure has a PDB code 2KHO; negatively charged PS molecules are shown in red.

To evaluate the effect of PS fatty acid chains on Hsp70-induced pore formation, besides POPS, characterizing by one saturated palmitoyl (16:0) tail and one unsaturated oleoyl (18:1) chain, bilayers composed of symmetrical DOPS, having two oleoyl tails, were also examined. The addition of Hsp70 to membranes composed of DOPS did not cause the appearance of step-like current fluctuations, only single detergent-like fluctuations of irregular profile were observed (Fig. 6c). The threshold concentration of protein for disintegration DOPS bilayers was about 4.4 µg/mL. These finding may point that Hsp70 could interact to DOPS but cannot form stable ion-permeable pores. This might be explained in the terms of pivotal role of membrane thickness and/or saturation of PS acyl chains in pore-forming ability of the protein. The hydrophobic regions which are thought to interact to membrane lipids were found at the C-terminus of the NBD^70^ and N-terminus of the SBD of Hsp70^71^. The possible dependence on the bilayer thickness is in a good agreement to an assumption of Hsp70-induced interdigitation (Fig. 7). Probably, the protein is able to form pores in membranes of defined thickness (with ≤ 16 hydrocarbon units), while an increase in the acyl tail length (up to 18) would inhibit this type of the protein activity. Hsp70-induced interdigitation might decrease hydrophobic mismatch and promote a formation of Hsp70 pores. Lipids with two unsaturated tails such as DOPS cannot be interdigitated by chemical inducers^35^, and Hsp70 cannot adjust the surrounding lipid media to hydrophobic thickness of its channels. Figure 6d demonstrates that Hsp70 also induced the step-like current fluctuations corresponding to openings and closures of single protein-induced pores in the membranes composed of DPhPS. This lipid characterized by two saturated 16:0 tails with four methyl branches (4CH_3_) in each other. The formation of pores in DPhPS bilayers at the same protein concentration range as in POPS bilayers does not confirm the hypothesis of specific interaction of Hsp70 with straight saturated (palmitoyl) chain, as methyl radicals should prevent the interaction. But these data do not contradict to the assumption of suitability of length of phytanoyl tails to hydrophobic length of Hsp70 channel. Moreover, the results of measuring the release of the fluorescent marker calcein from liposomes enriched with various PSs were in good agreement with the speculation that the protein is unable to form pores in membranes made from DOPC and DOPS (Supplementary Table S3).

Presumably, the ability of Hsp70 to form pores in the plasma membrane may play a role in the transport of various molecules (peptides, proteins, miRNAs) into or out of the cell by regulating the proper ionic environment. For example, Gross et al.^72^ proposed that penetration of the serine protease granzyme B into mHsp70-positive tumor cells is due to the formation of selective cation channels that facilitate the binding and absorption of granzyme B. In the absence of Hsp70 in the plasma membrane, these channels cannot be created and thus uptake of granzyme B is prohibited. The particular entry of granzyme B into mHsp70-positive cells mediates apoptosis in a perforin-independent manner^73,74^.

## Conclusions

The present study confirmed the ability of Hsp70 to form ion-permeable pores in PS-enriched membranes, probably depending on the bilayer thickness. Moreover, we have shown for the first time that the chaperone individually influences the membrane domain organizations and the thermotropic characteristics of saturated PS, leading to the formation of quasi-interdigitated lipid phase. Certainly, this process might play a role in reducing the sensitivity of cancer cells to radiation and chemotherapy due to altered membrane rigidity. However, to clarify the pattern of interdigitation, small- and wide-angle X-ray scattering diffraction experiments are required. It should be noted that the described results were obtained for a recombinant human Hsp70 without taking into account Mg^2+^, Ca^2+^, K^+^ ions, ATP/ADP nucleotides, and co-chaperones, which make a significant contribution to the functioning of the protein under physiological conditions. Finally, detailed analysis of the possibility and the role in tumor pathogenesis of induction of interdigitated lipid domains in cell membranes by Hsp70 might reveal the mechanisms of cancer cell resistance and promote to the development of new therapeutic target agents.

## Methods

### Chemicals

All chemicals were of reagent grade. Synthetic 1-palmitoyl-2-oleoyl-*sn*-glycero-3-phosphocholine (POPC), 1-palmitoyl-2-oleoyl-*sn*-glycero-3-phospho-l-serine (POPS), 1,2-dioleyl-*sn*-glycero-3-phospho-L-serine (DOPS), 1,2-diphytanoyl-*sn*-glycero-3-phospho-L-serine (DPhPS), 1,2-dimyristoyl-*sn*-glycero-3-phosphocholine (DMPC), 1,2-dimyristoyl-*sn*-glycero-3-phospho-l-serine (DMPS), 1,2-dipalmitoyl-*sn*-glycero-3-phosphocholine (DPPC), 1,2-dipalmitoyl-*sn*-glycero-3-phospho-l-serine (DPPS), 1,2-dipalmitoyl-*sn*-glycero-3-phospho-(1′-rac-glycerol) (DPPG), 1′,3′-bis[1,2-dimyristoyl-*sn*-glycero-3-phospho]-glycerol (TMCL), and 1,2-dipalmitoyl-*sn*-glycero-3-phosphoethanolamine-*N*-(lissamine rhodamine B sulfonyl) (Rh-DPPE) were obtained from Avanti Polar Lipids, Inc. (Pelham, AL). Sorbitol, KCl, HEPES, KOH, calcein, Triton X-100, and Sephadex G-50 were purchased from Sigma-Aldrich Company Ltd. (Gillingham, United Kingdom), phosphate buffered saline (PBS) was supplied by Rosmedbio (St. Petersburg, Russia). Solutions of 0.1 M KCl were buffered using 5 mM HEPES–KOH at pH 7.4.

### Recombinant human Hsp70

Recombinant human Hsp70 was obtained from *E. coli* transformed with a pMSHSP plasmid^75^. Briefly, chaperone was purified with anion exchange chromatography employing DEAE-Sepharose (GE Healthcare, USA) with subsequent ATP-affinity chromatography using ATP-Agarose (Sigma-Aldrich, USA). Bacterial lipopolysaccharides (LPS) were depleted employing Polymyxin B-Agarose gel (Sigma) with the endotoxin content below 0.1 EU/mg Hsp70.

### Differential scanning microcalorimetry

Differential scanning microcalorimetry experiments were performed by a μDSC 7EVO microcalorimeter (Setaram, Caluire-et-Cuire, France). Giant unilamellar vesicles (GUVs) were prepared from pure DMPC, DMPS, DPPS, DPPG, TMCL and from mixture of DMPS/DPPS (50/50 mol.%) by the electroformation method using Vesicle Pre Pro^@^ (Nanion Technologies, Munich, Germany) (standard protocol, 3 V, 10 Hz, 1 h, 45 °C or 65 °C). The resulting liposome suspension contained 2 mM lipid and was buffered by 5 mM HEPES–KOH at pH 7.4. Hsp70 from 1 mg/mL PBS stock solution was added to aliquots at concentrations of 20, 40, 80, 110, 160, and 300 µg/mL (lipid:Hsp70 molar ratios were about 7200:1, 3600:1, 1800:1, 1350:1, 900:1, and 450:1 respectively). The liposomal suspension was heated and cooled at a constant rate of 0.2 and 0.3 °C/min, respectively. The reversibility of the thermal transitions was assessed by reheating the sample immediately after the cooling step from the previous scan. The temperature dependence of the excess heat capacity was analyzed using Calisto Processing (Setaram, Caluire-et-Cuire, France). The peaks on the thermograms were characterized by the maximum temperature of the main phase transition (*T*_*m*_) of DMPC, DMPS, DPPS or DMPS/DPPS (50/50 mol.%) the half-width of the main peak (*T*_*1/2*_), characterizing the inverse cooperativity of the melting, the enthalpy of the main phase transition (an area of the main peak, ∆*H*), and the difference in the transition temperatures between heating and cooling scans (∆*T*_*h*_).

### Confocal fluorescence microscopy of domain organization of giant unilamellar vesicles

GUVs were formed by the electroformation method on a pair of indium tin oxide slides with using a commercial Vesicle Pre Pro^@^ (Nanion Technologies, Munich, Germany) as previously described^76^. Lipid stock solutions of POPC/DPPS (80/20 mol.%) and POPC/POPS/DPPC (60/20/20 mol.%) were prepared in chloroform. Labeling was carried out by an addition of the fluorescent lipid probe, Rh-DPPE; its concentration in each sample was 1 mol.%. Rh-DPPE clearly favors liquid disordered phase (*L*_*α*_) and it is excluded from ordered phase (*S*_*o*_)^62^. The resulting aqueous liposome suspension containing 1 mM lipid and 0.5 M sorbitol was divided into 10 μL aliquots. Hsp70 from 1 mg/mL PBS stock solution was added to aliquots at concentration of 140 µg/mL (the lipid:Hsp70 molar ratio was about 500:1). The liposome suspension with Hsp70 was allowed to equilibrate for 30 min at room temperature (25 ± 1 °C). The sample was observed as a standard microscopy preparation. 10 μL of the resulting liposome suspension without and with Hsp70 was placed on a standard microscope slide and covered by a cover slip. Vesicles were imaged through an oil immersion objective (PLAPON60XO, 60 ×/1.42 NA) using a confocal laser scanning microscope Olympus FV3000 (Olympus, Tokyo, Japan). A helium–neon laser with a wavelength of 561 nm was used to excite Rh-DPPE. Temperature during observation was controlled by the air heating/cooling in the thermally insulated camera.

The percentage of vesicles (*p*_*i*_) with the respective phase separation type in each tested system was calculated as the ratio of phase-separated (coexistence of *L*_*α*_ and *S*_*o*_) or homogenous (*L*_*α*_ only) GUVs to the total number of GUVs:

1 $$p_{i} = \frac{{N_{i} }}{{N_{t} }} \cdot 100\% ,$$

where the *i*-type of the phase separation scenario in GUVs indicates a homogeneously colored GUVs in the *L*_*α*_-phase or liposomes with irregular uncolored *S*_*o*_ domains; *N*_*i*_—number of vesicles with the *i*-th type of the phase separation scenario (from 0 to 150); and *N*_*t*_—total number of counted vesicles in the sample (typically about 150). At least 10 independent experiments were performed with each lipid system.

To calculate the relative area of *S*_*o*_ domains in liposomes without and with Hsp70 (*A*_*r*_), the obtained images (150 ± 15) were processed using ImageJ software (NIH, Washington, USA).

### Registration of Hsp70 ion channels in planar lipid bilayers

Virtually solvent-free planar lipid bilayers were prepared according to a monolayer-opposition technique^77^ on a 50-µm-diameter aperture in a 10-µm thick Teflon film separating two (*cis* and *trans*) compartments of the Teflon chamber. The aperture was pretreated with hexadecane. The lipid bilayers were made from pure POPC, POPS, DOPS, or DPhPS. After the membrane was completely formed and stabilized, Hsp70 from a stock solution (1 mg/mL in PBS) was added to *cis*-compartments to obtain concentrations in the range from 0.1 to 6.0 µg/mL. All experiments were performed at room temperature (of 20 °C).

Ag/AgCl electrodes with 1.5% agarose/2 M KCl bridges were used to apply *V* and measure the transmembrane current (*I*). “Positive voltage” refers to the case in which the *cis*-side compartment is positive with respect to the *trans*-side. Transmembrane current was measured using an Axopatch 200B amplifier (Molecular Devices, LLC, Orleans Drive, Sunnyvale, CA, USA) in the voltage clamp mode. Data were digitized using a Digidata 1440A and analyzed using pClamp 10.0 (Molecular Devices, LLC, Orleans Drive, Sunnyvale, CA, USA) and Origin 8.0 (OriginLab Corporation, Northampton, MA, USA). Data were acquired at a sampling frequency of 5 kHz using low-pass filtering at 1 kHz, and the current tracks were processed through an 8-pole Bessel 100-kHz filter.

Each lipid system was characterized by the threshold concentration of Hsp70 required to appearance of the transmembrane ion-permeable pores (*C*_*sc*_) and the threshold concentration of Hsp70 required to reach the disintegration of the lipid bilayers (*C*_*tr*_).

### Calcein release from large unilamellar vesicles

Large unilamellar vesicles were prepared from pure POPC and DOPS, and a mixture of POPC/POPS (50/50 mol.%) and loaded with the fluorescent dye calcein (35 mM calcein, 10 mM HEPES–KOH, pH 7.4) by extrusion as previously described^78^. At this concentration calcein fluorescence inside the liposomes is self-quenched. Hsp70 from a stock solution (1 mg/mL in PBS) was added to calcein-loaded liposomes in concentration of 142 × 10^−9^ M (10 µg/mL), corresponding to the lipid:protein molar ratio of about 1300:1 (similar to the molar ratio used in the electrophysiological experiments). Time-dependence of calcein fluorescence de-quenching induced by 10 µg/mL of Hsp70 was measured over 20 min at 25 °C. The degree of calcein release was determined using a Fluorat-02-Panorama spectrofluorimeter (Lumex, Saint-Petersburg, Russia). The excitation wavelength was 490 nm and the emission wavelength was 520 nm.

The relative intensity of calcein fluorescence (*RF*, %) was calculated using the following formula:

2 $$RF= \frac{I-{I}_{0}}{\frac{{I}_{max}}{0.9}-{I}_{o}}\cdot 100\%$$

where *I* and *I*_*0*_—calcein fluorescence intensity of the sample in the presence and absence of protein, respectively, and *I*_*max*_—maximal fluorescence of the sample after lysis of all liposomes by Triton X-100. Factor of 0.9 was introduced in order to take into account a dilution of the sample by Triton X-100.

The characteristic parameters of calcein release from liposomes in the presence of protein were averaged from 2–3 experiments for each system (mean ± sd, p ≤ 0.05).

### Statistics and reproducibility

The data were graphed and analyzed using Prism GraphPad 8 (GraphPad Software, CA, USA). The values of *C*_*sc*_, *C*_*tr*_, Δ*T*_*m*_, Δ*T*_*1/2*_, ΔΔ*T*_*h*_, and ΔΔ*H* were averaged from 2 to 4 independent experiments and presented as *mean* ± *SE* (*p* ≤ 0.05). The values of *p*_*i*_ and *Ar* were averaged from at least 10 independent experiments and represent as *mean* ± *SD* (*p* ≤ 0.05). The data was verified for normality using the Shapiro–Wilk and Kolmogorov–Smirnov tests. The statistical significance was evaluated by paired two-tailed *t* test. The significance was represented as ^*ns*^*p* ≥ 0.05, *p ≤ 0.05, **p ≤ 0.01, ***p ≤ 0.001, and ****p ≤ 0.0001.

### Reporting summary

Further information on research design is available in the Nature Portfolio Reporting Summary linked to this article.

### Supplementary Information

Supplementary Information.

## Data Availability

The datasets used and/or analysed during the current study are available from the corresponding author Dr. Maxim Shevtsov on reasonable request.

## Abbreviations

DMPC: 1,2-Dimyristoyl-*sn*-glycero-3-phosphocholine
DMPS: 1,2-Dimyristoyl-*sn*-glycero-3-phospho-l-serine
DOPS: 1,2-Dioleyl-*sn*-glycero-3-phospho-L-serine
DPPC: 1,2-Dipalmitoyl-*sn*-glycero-3-phosphocholine
DPPS: 1,2-Dipalmitoyl-*sn*-glycero-3-phospho-L-serine
DPhPS: 1,2-Diphytanoyl-*sn*-glycero-3-phospho-L-serine
DPPG: 1,2-Dipalmitoyl-*sn*-glycero-3-phospho-(1′-rac-glycerol)
TMCL: 1′,3′-Bis[1,2-dimyristoyl-*sn*-glycero-3-phospho]-glycerol
GUV: Giant unilamellar vesicle
Hsp70: Heat shock protein 70 kDa
NBD: Nucleotide-binding domain
PC: Phosphatidylcholine
POPC: 1-Palmitoyl-2-oleoyl-*sn*-glycero-3-phosphocholine
POPS: 1-Palmitoyl-2-oleoyl-*sn*-glycero-3-phospho-l-serine
PS: Phosphatidylserine
Rh-DPPE: 1,2-Dipalmitoyl-*sn*-glycero-3-phosphoethanolamine-*N*-(lissamine rhodamine B sulfonyl)
SBD: Substrate-binding domain
