# Supplementary material for: A new look at Hsp70 activity in phosphatidylserine-enriched membranes: chaperone-induced quasi-interdigitated lipid phase
Source: Sci Rep. 2023 Nov 6;13:19233. doi: 10.1038/s41598-023-46131-x (PMC10628215; doi:10.1038/s41598-023-46131-x)
Supplement: Supplementary file 1 — Supplementary Information. [file 41598_2023_46131_MOESM1_ESM.docx]

**Supplementary information**

**Table S1.** Thermothropic characteristics of phosphatidylserines in the presence of Hsp70.

| **Liposome composition** | **Hsp70 concentration, μg/mL** | **Thermothropic characteristics** | | | |
| --- | --- | --- | --- | --- | --- |
|  |  | **Δ*T_m_*, °C** | **Δ*T_1/2_*, °C** | **ΔΔ*T_h_*, °C** | **ΔΔ*H*, kcal/mol** |
| DMPS | 80 | -0.4 ± 0.1 | 0.4 ± 0.3 | 0.1 ± 0.1 | 0.2 ± 0.1 |
|  | 110 | -0.1 ± 0.1 | 0.4 ± 0.1 | 0.2 ± 0.1 | 0.5 ± 0.2 |
|  | 160 | 0.2 ± 0.1 | 0.6 ± 0.4 | 0.2 ± 0.1 | 0.6 ± 0.2 |
|  | 300 | 0.3 ± 0.1 | 0.7 ± 0.2 | 0.3 ± 0.1 | 0.5 ± 0.2 |
| DPPS | 80 | 0.1 ± 0.1 | 0.7 ± 0.2 | 0.5 ± 0.1 | 0.4 ± 0.2 |
|  | 110 | 0.5 ± 0.2 | 0.9 ± 0.1 | 0.5 ± 0.2 | 0.6 ± 0.3 |
|  | 160 | 0.9 ± 0.2 | 1.0 ± 0.1 | 0.6 ± 0.2 | 0.7 ± 0.2 |
|  | 300 | 1.0 ± 0.1 | 1.1 ± 0.2 | 0.6 ± 0.1 | 0.6 ± 0.3 |
| DMPS/DPPS  (50/50 mol.%) | 80 | 0.5 ± 0.1 | 0.3 ± 0.2 | 0.2 ± 0.1 | 2.8 ± 0.5 |
|  | 110 | 0.8 ± 0.2 | 0.7 ± 0.1 | 0.3 ± 0.1 | 4.3 ± 0.4 |
|  | 160 | 1.1 ± 0.1 | 0.9 ± 0.1 | 0.3 ± 0.1 | 4.9 ± 0.6 |
|  | 300 | 1.4 ± 0.2 | 1.0 ± 0.1 | 0.3 ± 0.1 | 5.2 ± 0.7 |

Δ*T*_m_, Δ*T*_1/2_ – the changes in the melting temperature of DMPS, DPPS, and DMPS/DPPS (50/50 mol.%) and the half-width of the transition peak. The *T_m_* of protein-untreated DMPS, DPPS, and DMPS/DPPS (50/50 mol.%) was equal to 36.9 ± 0.1, 53.2 ± 0.2, and 46.7 ± 0.2°C respectively. *T_1/2_* of unmodified liposomes was equal to 1.1 ± 0.1, 1.2 ± 0.2, and 2.2 ± 0.1°C respectively;

ΔΔ*T_h_* – the changes in the difference in the transition temperatures between heating and cooling scans (alteration in *T_m_*-hysteresis). In the absence of Hsp70, Δ*T_h_* was equal to 0.6 ± 0.2, 0.9 ± 0.2, and 0.7 ± 0.1°C for DMPS, DPPS, and DMPS/DPPS (50/50 mol.%) respectively;

ΔΔ*H* – the changes in the transition enthalpy. Δ*H* of protein-untreated DMPS, DPPS, and DMPS/DPPS (50/50 mol.%) was equal to 7.5 ± 0.1, 11.9 ± 0.3, and 2.9 ± 0.9 kcal/mol respectively.

**Figure S1.** Heating thermograms of DPPG (**a**) and TMCL (**b**) in the absence (control, *black curves*) and presence of Hsp70 at concentration of 20 (*orange* *curves*), 40 (*violet* *curves*), 80 (*red curves*), 110 (*green curves*), 160 (*blue curves*) and 300 μg/mL (*cyan curves*).

**Table S2.** Thermothropic characteristics of various anionic lipids in the presence of Hsp70.

| **Liposome composition** | **Hsp70 concentration, μg/mL** | **Thermothropic characteristics** | | | |
| --- | --- | --- | --- | --- | --- |
|  |  | **Δ*T_m_*, °C** | **Δ*T_1/2_*, °C** | **ΔΔ*T_h_*, °C** | **ΔΔ*H*, kcal/mol** |
| DPPG | 20 | -0.1 ± 0.1 | 0 | 0.1 ± 0.1 | 0 |
|  | 40 | -0.1 ± 0.1 | 0 | 0.1 ± 0.1 | 0 |
|  | 80 | -0.1 ± 0.1 | 0.1 ± 0.1 | 0.1 ± 0.1 | 0.1 ± 0.1 |
|  | 110 | 0 | 0.1 ± 0.1 | 0.2 ± 0.1 | 0.1 ± 0.1 |
|  | 160 | 0.1 ± 0.1 | 0.1 ± 0.1 | 0.2 ± 0.1 | 0.2 ± 0.1 |
|  | 300 | 0.1 ± 0.1 | 0.1 ± 0.1 | 0.3 ± 0.1 | 0.2 ± 0.1 |
| TMCL | 20 | 0 | 0 | 0.1 ± 0.1 | 0.2 ± 0.1 |
|  | 40 | 0 | 0 | 0.1 ± 0.1 | 0.2 ± 0.1 |
|  | 80 | 0.1 ± 0.1 | 0 | 0.1 ± 0.1 | 0.2 ± 0.1 |
|  | 110 | 0.2 ± 0.1 | 0.1 ± 0.1 | 0.2 ± 0.1 | 0.2 ± 0.2 |
|  | 160 | 0.2 ± 0.1 | 0.1 ± 0.1 | 0.2 ± 0.1 | 0.3 ± 0.1 |

Δ*T*_m_, Δ*T*_1/2_ – the changes in the melting temperature of DPPG and TMCL and the half-width of the transition peak. The *T_m_* of protein-untreated DPPG and TMCL was equal to 41.1 ± 0.2 and 44.9 ± 0.4°C respectively. *T_1/2_* of unmodified liposomes was equal to 0.8 ± 0.1 and 1.2 ± 0.2°C respectively;

ΔΔ*T_h_* – the changes in the difference in the transition temperatures between heating and cooling scans (alteration in *T_m_*-hysteresis). In the absence of Hsp70, Δ*T_h_* was equal to 0.7 ± 0.1 and 0.9 ± 0.1°C for DPPG and TMCL respectively;

ΔΔ*H* – the changes in the transition enthalpy. Δ*H* of protein-untreated DPPG and TMCL was equal to 10.5 ± 0.2 and 8.3 ± 0.7 kcal/mol respectively.

**Table S3.** The calcein release from large unilamellar vesicles induced by 10 µg/mL of Hsp70 (*RF*, %).

| **Lipid composition** | ***RF_max_*, %** |
| --- | --- |
| POPC | 2 ± 2 |
| DOPS | 3 ± 2 |
| POPC/POPS (50/50 mol.%) | 24 ± 5 |
